# Clinicopathological Spectrum of Primary and Metastatic Neuroendocrine Neoplasms

**DOI:** 10.7759/cureus.11764

**Published:** 2020-11-29

**Authors:** Atif A Hashmi, Javaria Ali, Kiran Khan, Omer Ahmed, Ata ur Rehman, Muhammad Irfan, Saroona Haroon, Muhammad Ghani Asif

**Affiliations:** 1 Pathology, Liaquat National Hospital and Medical College, Karachi, PAK; 2 Community Health Sciences, Hamdard College of Medicine and Dentistry, Karachi, PAK; 3 Internal Medicine, Liaquat National Hospital and Medical College, Karachi, PAK; 4 Pharmacology and Therapeutics, Hamdard College of Medicine and Dentistry, Karachi, PAK; 5 Statistics, Liaquat National Hospital and Medical College, Karachi, PAK; 6 Pathology, Prince Faisal Oncology Centre, King Fahad Specialist Hospital, Buraidah, SAU; 7 Pathology, Multan Medical and Dental College, Multan, PAK

**Keywords:** neuroendocrine tumors, well differentiated neuroendocrine tumor, poorly differentiated neuroendocrine carcinoma, carcinoid, atypical carcinoid, tumor grade

## Abstract

Introduction

Neuroendocrine neoplasms (NENs) are a heterogeneous group of tumors with histological features varying from well-differentiated neuroendocrine tumors (WDNETs) to poorly differentiated neuroendocrine carcinomas (PDNECs). In this study, we investigated the clinicomorphological spectrum of NENs including tumor grade, site of origin, and metastasis.

Methods

We retrospectively studied 125 cases of NENs (at the Department of Histopathology, Liaquat National Hospital and Medical College, Karachi) between the years 2014 and 2020. Slides of these cases were retrieved from the departmental archives and were evaluated for the tumor type, grade, and site of origin.

Results

The mean age of the patients was 51.25±16.10 years. Overall, the liver was the most common site of the tumor (27.2%), followed by the small bowel (15.2%). Grade 2 was the most common tumor grade (40.8%), and most of the tumors were primary (68.8%). A total of 84.8% of the tumors were WDNETs/carcinoids, while 15.2% were PDNEC. The small bowel was the most common site of primary NENs, followed by the stomach and lung. Among primary neuroendocrine tumors, patients with PDNEC were significantly noted to have a higher mean age than WDNET/carcinoid. Similarly, PDNEC had a higher ki67 index than WDNET/carcinoid. For metastatic NENs, the liver was the most common site of metastasis (71.8%) with the GI/pancreatobiliary tract being the most common primary site of origin (51.3%). Tumors with primary lung origin were found to have a higher tumor grade than primary GI/pancreatobiliary tract origin NENs (p<0.0001).

Conclusion

In this study, we found that the small intestine and liver were the most common sites for primary and metastatic NENs, respectively. Moreover, primary PDNECs were associated with a higher mean age than WDNETs. Alternatively, metastatic NENs with primary lung origin had a higher tumor grade than primary GI/pancreatobiliary tract origin.

## Introduction

Neuroendocrine neoplasms (NENs) are a heterogeneous group of tumors with neuronal and endocrine differentiation while having a common phenotype [1]. While having a diverse pattern of histological features varying from well-differentiated neuroendocrine tumors (WDNETs) to poorly differentiated neuroendocrine carcinomas (PDNECs), their pathogenesis has been elaborated in various studies previously [2].

By virtue of the body-wide distribution of neuroendocrine cells, NENs have been observed in almost all anatomical locations, such as the GI tract, respiratory tract, central nervous system, thyroid, skin, breast, and urogenital system [3]. However, the most common sites for primary NENs include the GI tract, pancreatobiliary tract, and lungs [4,5].

NENs, in general, share the expression of common neuroendocrine markers such as synaptophysin and chromogranin A. However, due to diverse histomorphological features and clinical aggressiveness, their classification varies from organ to organ, depending upon the grade and site of origin.

The latest edition of the WHO classifies the GI NENs into grades G1, G2, and G3 based on mitotic activity, Ki-67 labeling index, and the presence of necrosis. Neuroendocrine carcinomas by definition are considered high grade [6]. In contrast, lung NENs are grouped into four histologic variants, namely, typical carcinoid (TC), atypical carcinoid (AC), large cell neuroendocrine carcinoma (LCNEC), and small cell neuroendocrine carcinoma (SCNEC) [7].

The aggressiveness of NENs varies, primarily based on the site of origin. Generally, NENs of the small intestine have a high malignant potential, but they progress indolently in case of metastasis. Conversely, gastric and rectal NENs have a low tendency for metastasis but can progress rapidly once they become metastatic.

In our study, we explored the clinicomorphological spectrum of NENs including tumor grade, site of origin, and metastasis.

## Materials and methods

We retrospectively studied 125 cases of NENs (at the Department of Histopathology, Liaquat National Hospital and Medical College, Karachi) between the years 2014 and 2020. Slides of these cases were retrieved from the departmental archives and were evaluated for the tumor type, grade, and site of origin. Immunohistochemical (IHC) stains were performed on all cases of primary and metastatic NENs. For primary NENs, pan-cytokeratin (CKAE1/AE3), synaptophysin, chromogranin A and Ki67 immunostains were performed. For metastatic NENs. In addition to these above-mentioned stains, a panel of additional IHC stains, including CK7, CK20, CDX2 (a marker of GI/pancreatobiliary tract origin), and TTF1 (a marker of lung origin) were performed to determine the primary site of origin. The final primary site of origin for metastatic tumors was determined considering IHC results with clinical and radiologic correlation.

**Figure 1:**
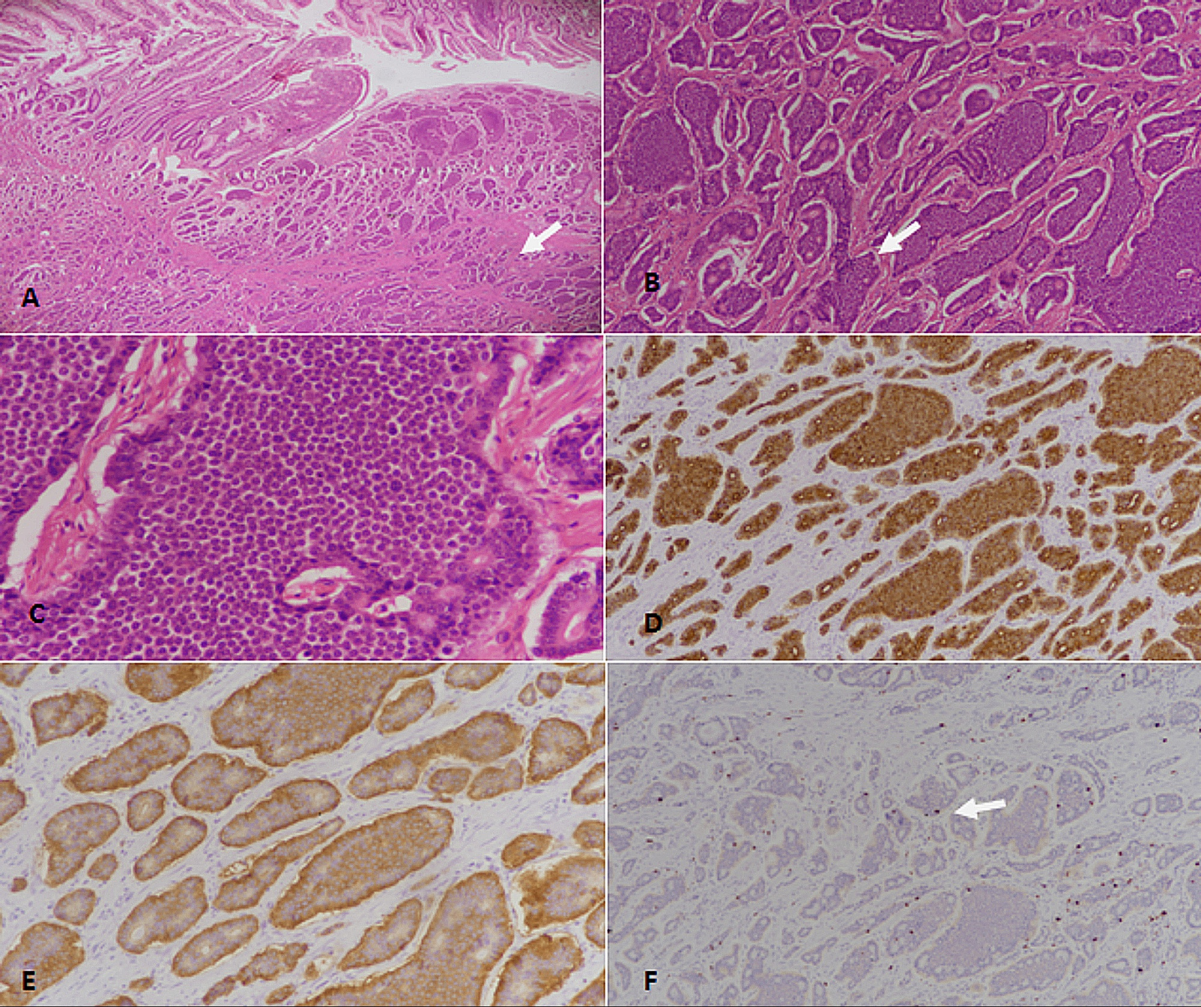
Neuroendocrine tumor grade 1 of primary gastrointestinal tract origin. (A) H&E stained sections at 40× magnification showing nests and clusters of NET in the mucosa and submucosa (arrow). (B) 100× magnification showing nests and clusters of NET (arrow). (C) 400× magnification depicting tumor cells with stippled chromatin and lack of mitotic activity. (D) Pan-cytokeratin (CKAE1/AE3) immunostain revealing diffuse positivity in tumor cells. (E) Synaptophysin immunostain showing diffuse cytoplasmic positivity in tumor cells. (F) Ki67 immunostain revealing <2% proliferative index (arrow showing nuclear staining in occasional tumor cells). H &E: hematoxylin and eosin; NET: neuroendocrine tumor

Morphologically, WDNETs have cells arranged in islets, ribbons, trabeculae, organoids, or rosette formations. Individual tumor cells appear monomorphous, with a round to oval nuclei with salt and pepper chromatin and inconspicuous nucleoli, along with moderate to abundant eosinophilic cytoplasm (Figure 1).

**Figure 2:**
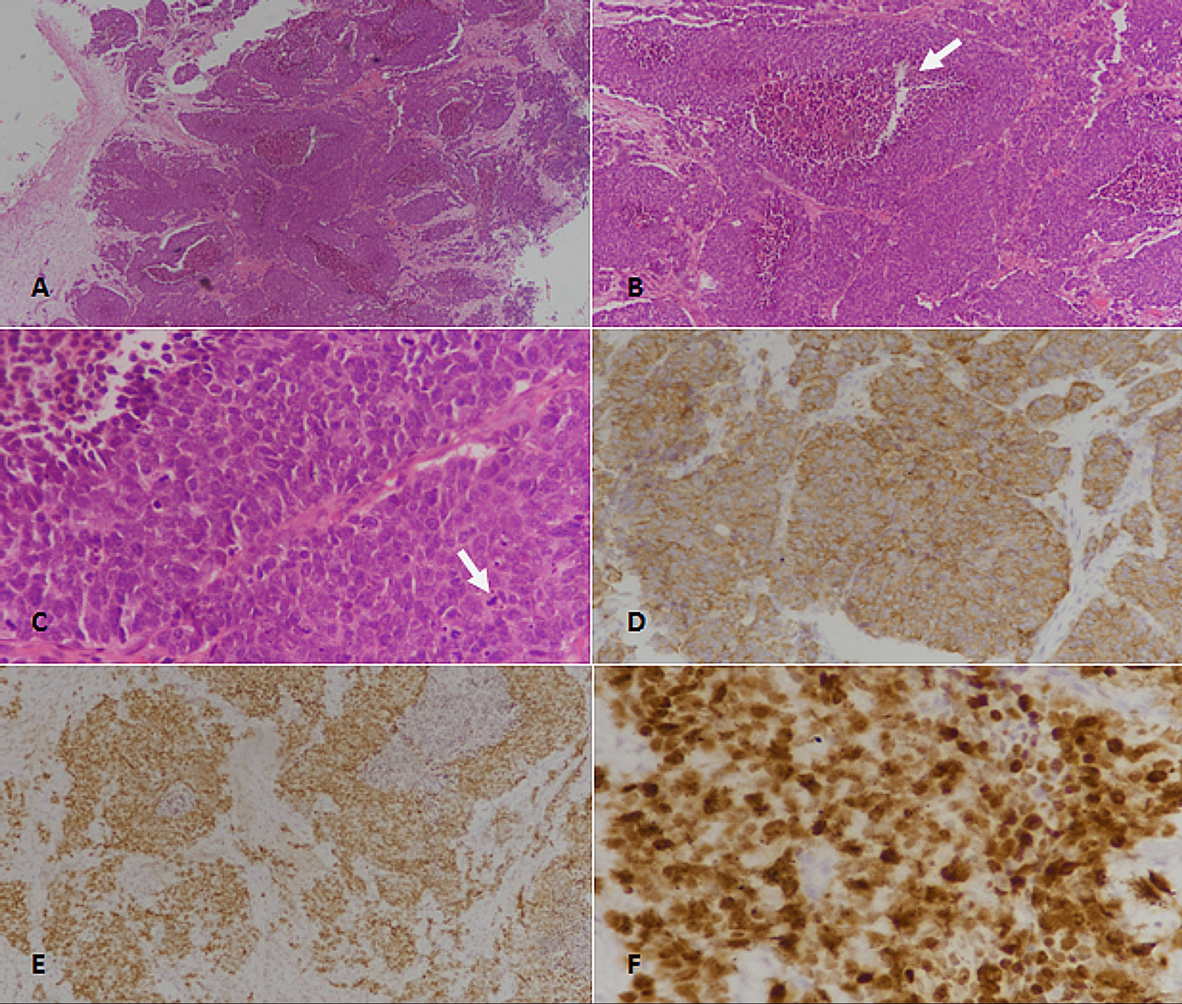
Poorly differentiated neuroendocrine carcinoma of primary lung origin (large cell neuroendocrine carcinoma of the lung). (A) H&E sections at 40× magnification showing large sheets of tumor cells. (B) 100× magnification revealing sheets of tumor cells with areas of necrosis (arrow). (C) 400× magnification showing large-sized tumor cells with frequent atypical mitoses (arrow). (D) Synaptophysin immunostain depicting cytoplasmic and membranous positivity in tumor cells. (E) TTF1 immunostain revealing nuclear immunoreactivity. (F) Ki67 immunostain showing high (>90%) proliferative index. H &E: hematoxylin and eosin, TTF1: thyroid transcription factor 1

In contrast, PDNECs have a sheet-like arrangement of pleomorphic cells with variable areas of necrosis. PDNEC were further sub-classified into SCNEC and LCNEC. SCNEC had fusiform nuclei, tightly packed overlapping nuclei with finely granular chromatin, while LENEC had more-rounded and atypical nuclei, sometimes with prominent nucleoli (Figure 2).

Tumor grade was defined numerically into three grades based on the number of mitosis per 10 high power fields (HPFs) and Ki-67 index: G1 tumors had a mitotic rate from zero to one per 10 HPFs or a Ki‐67 index from 0% to 2%, G2 tumors had a mitotic rate from two to 20 per 10 HPFs or a Ki‐67 index from 3% to 20%, and G3 tumors had a mitotic rate greater than 20 per 10 HPFs or a Ki‐67 index greater than 20% [8]. Both mitosis and Ki-67 index were observed in the most mitotically active areas of the tumor as previous studies showed a considerable degree of intra-tumoral heterogeneity [9].

Data analysis was performed using Statistical Package for Social Sciences (Version 26.0, IBM Inc., Armonk, USA). Independent t-test, one-way analysis of variance (ANOVA), and Fisher's exact test were used to check the association. P-values ≤ 0.05 were considered as significant.

## Results

The mean age of the patients was 51.25±16.10 years. And, 59.2% of patients were male. Overall, the liver was the most common site of the tumor (27.2%), followed by the small bowel (15.2%). Grade 2 was the most common tumor grade (40.8%), and most of the tumors were primary (68.8%). A total of 84.8% of the tumors were WDNETs/carcinoids, while 15.2% were PDNEC (Table 1).

**Table 1:** Overall characteristics of the study population (n = 125) *Mean±SD. **Gastrointestinal/pancreatobiliary tract origin neuroendocrine tumors are called well-differentiated neuroendocrine tumors, while lung origin is referred to as carcinoids (typical/atypical).

Clinicopathological characteristics	N (%)
Age (years)*	51.25±16.10
Ki67 Index (%)*	31.32±32.43
Gender	
Male	74(59.2)
Female	51(40.8)
Site	
Liver	34(27.2)
Rectum	6(4.8)
Stomach	17(13.6)
Small Bowel	19(15.2)
Lung	17(13.6)
Appendix	6(4.8)
Anal Canal	3(2.4)
Esophagus	2(1.6)
Colon	3(2.4)
Pancreas	7(5.6)
Mediastinum	2(1.6)
Mesentery	2(1.6)
Gall Bladder	4(3.2)
Urinary Bladder	1(0.8)
Brain	2(1.6)
Grade	
Grade 1	29(23.2)
Grade 2	51(40.8)
Grade 3	45(36)
Primary vs. Metastatic	
Primary	86(68.8)
Metastatic	39(31.2)
Type of Neuroendocrine Tumor	
Well-Differentiated Neuroendocrine Tumor/Carcinoid**	106(84.8)
Poorly Differentiated Neuroendocrine Carcinoma	19(15.2)

The mean age of the patients with primary NENs was 49.40±17.10 years and most of the patients were male (64%). The small bowel was the most common site of primary NENs, followed by the stomach and lung. Among primary neuroendocrine tumors, patients with PDNC were significantly noted to have a higher mean age than WDNET/carcinoid. Similarly, PDNEC had a higher ki67 index than WDNET/carcinoid. Although PDNECs were seen in higher numbers in the lung, rectum, and pancreas, the difference was not statistically significant (Table 2).

**Table 2:** Clinicopathological features of primary neuroendocrine tumors and association of clinicopathological features with type of neuroendocrine tumor (n = 86) *Mean±SD, Independent t-test was applied. **Fisher's exact test was applied. ***Gastrointestinal/pancreatobiliary tract origin neuroendocrine tumors are called well-differentiated neuroendocrine tumors, while lung origin is referred to as carcinoids (typical/atypical). ****p-Value significant as <0.05.

Clinicopathological characteristics	Type of neuroendocrine tumor n (%)			p-Value
	Overall	Well-differentiated neuroendocrine tumor/Carcinoid***	Poorly differentiated neuroendocrine carcinoma	
Age (years)*	49.40±17.10	47.31±16.53	67.33±10.39	0.001****
Ki67 (%)*	24.98±30.10	18.88±25.20	77.22±13.01	<0.0001****
Gender**				
Male	55(64)	50(64.9)	5(55.6)	0.717
Female	31(36)	27(35.1)	4(44.4)	
Site**				
Liver	6(7)	6(7.8)	0(0)	0.155
Rectum	6(7)	5(6.5)	1(11.1)	
Stomach	16(18.6)	16(20.8)	0(0)	
Small Bowel	19(22.1)	18(23.4)	1(11.1)	
Lung	16(18.6)	10(13)	6(66.7)	
Appendix	6(7)	6(7.8)	0(0)	
Anal Canal	3(3.5)	3(3.9)	0(0)	
Colon	3(3.5)	3(3.9)	0(0)	
Pancreas	7(8.1)	6(7.8)	1(11.1)	
Mediastinum	1(1.2)	1(1.3)	0(0)	
Gall Bladder	1(1.2)	1(1.3)	0(0)	
Urinary Bladder	1(1.2)	1(1.3)	0(0)	
Brain	1(1.2)	1(1.3)	0(0)	

Table 3 shows the clinicopathological features of metastatic neuroendocrine tumors. The mean age of the patients with metastatic NENs was 55.33±12.95 and the most common gender was female (51.3%). The liver was the most common site of metastasis (71.8%) with the GI/pancreatobiliary tract being the most common primary site of origin (51.3%). Metastatic tumors with primary lung origin were found to have a higher tumor grade than primary GI/pancreatobiliary tract origin NENs (p<0.0001). Alternatively, no significant association of grade was noted with respect to age, gender, or site of metastasis.

**Table 3:** Clinicopathological features of metastatic neuroendocrine tumors and association of clinicopathological features with tumor grade (n = 39) *Mean±SD, ANOVA was applied. **Fisher's exact test was applied. ***p-Value significant as <0.05.

Clinicopathological characteristics	Tumor grade				p-Value
	Overall	Grade 1	Grade 2	Grade 3	
Age (years)*	55.33±12.95	67.00±20.54	54.09±11.53	53.95±11.74	0.165
Gender**					
Male	19(48.7)	1(25)	6(54.5)	12(50)	0.791
Female	20(51.3)	3(75)	5(45.5)	12(50)	
Site**					
Liver	28(71.8)	2(50)	9(81.8)	17(70.8)	0.388
Stomach	1(2.6)	0(0)	0(0)	1(4.2)	
Lung	1(2.6)	0(0)	1(9.1)	0(0)	
Esophagus	2(5.1)	0(0)	0(0)	2(8.3)	
Mediastinum	1(2.6)	0(0)	1(9.1)	0(0)	
Mesentery	2(5.1)	1(25)	0(0)	1(4.2)	
Gall Bladder	3(7.7)	1(25)	0(0)	2(8.3)	
Brain	1(2.6)	0(0)	0(0)	1(4.2)	
Primary site of origin**					
Gastrointestinal/pancreatobiliary tract	20(51.3)	4(100)	10(90.9)	6(25)	<0.0001***
Lung	19(48.7)	0(0)	1(9.1)	18(75)	

## Discussion

In this study, we evaluated the clinicopathological characteristics of both primary and metastatic NENs. We found that the small bowel was the most common site of primary NENs. Primary PDNEC had a higher mean age than primary WDNT/carcinoid. Alternatively, for metastatic NENs, the liver was found to be the most common site of metastasis. In addition, we noted that metastatic lung NENs had significantly higher tumor grades than the metastatic GI/pancreatobiliary tract NENs.

WDNETs have an indolent behavior, but the tumor cells acquire traits facilitating metastasis. Although the malignant potential of small bowel NENs is directly proportional to tumor size, even sub-centimeter tumors have the metastatic potential [10]. Tumor grade itself plays an important role in predicting metastasis.

Determining the behavior of metastatic NENs is important as this in turn directs the course of treatment for these patients. Keck et al. in 2017 studied 103 patients with primary and metastatic NENs [11]. Their results showed that one-third of patients had metastasis with a different grade than their primary, and therefore determining the grade in both the primary tumor and metastasis is important for estimating prognosis. In our study, the majority of the metastatic tumors had high grade (61.5%).

By virtue of its rich dual blood supply, the liver appears to be the most common site for metastasis of various tumors including NENs as shown by previous studies [12]. It is crucial to locate the primary site for metastatic liver NETs in terms of prognosis and survival; however, in approximately 11-14% of cases, the primary site for metastatic liver NETs cannot be determined [13]. In our study, most of the cases of liver metastasis had a primary GI/pancreatobiliary tract origin, followed by the lung.

Formerly, WDNETs were graded into G1 and G2 only, while G3 NENs were classified as PDNECs. However, it is now clear that WDNETs can have all the three grades ranging from G1 to G3. The main distinction between WDNET and PDNEC is morphological architecture. WDNETs have an organoid or nested architecture on morphology, while PDNEC have a sheet-like architecture. The reason for this sharp separation between WDNET and PDNEC is that PDNEC have a distinct molecular pathway of origin, that is entirely different from WDNET and hence an entirely different prognosis [14]. Another update in the latest WHO classification of digestive tract NENs was regarding mixed neuroendocrine-non neuroendocrine neoplasms (MiNENs), previously called mixed adenocarcinoma neuroendocrine carcinoma (MANEC). This change was introduced based on the fact that the non-neuroendocrine component in a mixed tumor is not always an adenocarcinoma and the neuroendocrine component is not always a carcinoma (it can also be a WDNET) [15]. In our study, there was no MiNEN.

Our study had certain limitations. First, data regarding the clinical presentation of patients with NENs were not available. Second, the results of serological tests such as 5-hydroxy-indole-acetic acid (5-HIAA) and chromogranin A (CgA) were unobtainable. Moreover, clinical follow-up data were also unavailable. Due to the retrospective study design, confounding factors were not controlled. Despite these limitations, the study provides a foundation providing baseline clinicopathological features of NENs for future work considering all these limiting factors.

## Conclusions

NENs are an important category of tumors that can occur at any body site and therefore understanding the clinical and pathological behavior of NENs is crucial for both clinicians and pathologists. In this study, we found that the small intestine is the most common site for NENs. Primary PDNECs were found more likely to present at an older age than WDNETs. The liver was found to be the most common site for metastatic NENs with GI/pancreatobiliary tract as the most common primary site for these cases followed by the lung. We also noted in our study that tumors with primary lung origin were found to have a higher tumor grade than primary GI/pancreatobiliary tract origin NENs.
